# C5 palsy after C5/6/7 posterior foraminal decompression: A case report

**DOI:** 10.1097/MD.0000000000018817

**Published:** 2020-01-17

**Authors:** Masahito Oshina, Tomohide Segawa, Yasushi Oshima, Sakae Tanaka, Hirohiko Inanami

**Affiliations:** a Department of Orthopedic Surgery, Inanami Spine and Joint Hospital, 3-17-5, Higashishinagawa, Shinagawa-Ku; b Department of Orthopedic Surgery, The University of Tokyo Hospital 7-3-1, Hongo, Bunkyo-Ku, Tokyo, Japan.

**Keywords:** endoscopic, foraminotomy, palsy, radiculopathy, spine, surgical procedure

## Abstract

**Rationale:**

Although C5 palsy is a common complication of cervical spine surgery, its cause has not been confirmed. There are various hypotheses for its mechanism, including spinal cord impairment and nerve involvement. Therefore, prophylactic foraminotomy is one of the methods recommended for preventing C5 palsy. However, we describe a patient who experienced C5 palsy after microendoscopic foraminotomy between the left C5/6 and C6/7 levels.

**Patient concerns:**

A 43-year-old man presented with a 14-month history of progressive numbness in the left upper limb. We performed microendoscopic left foraminal decompressions at the C5/6/7 levels to treat the left C6 and C7 radiculopathy. On the postoperative day 1, we observed weak motor strength of the left deltoid, left biceps, and left forearm pronator, while the motor strength of the other muscles was normal.

**Diagnoses:**

C5 palsy following C5/6/7 left foraminotomy.

**Intervention:**

Follow-up rehabilitation with muscle strength training and range of motion training.

**Outcome:**

The patient recovered his motor strength completely within 3 months postoperatively.

**Lessons:**

In this case, the C5 palsy could not be adequately explained by the theory of nerve root impingement or disruption in blood circulation following spinal cord decompression. We hypothesized that the patient had drill heat-induced C5 palsy. Regarding the C5 palsy without C5 nerve root decompression, we hypothesize that the C5 palsy in C5/6/7 foraminotomy could be related to variations in the formation of the brachial plexus. Prophylactic foraminotomy for cervical posterior surgery should be performed with care, limiting its use in patients who are at a risk of developing C5 palsy because the prophylactic procedure can cause C5 palsy. We must also consider that even without decompression at the C4/5 level, there is a possibility of C5 palsy occurring.

## 1 Introduction

C5 palsy is a common complication of cervical spine surgery, with a reported incidence of 4.7% to 6.3%.^[1–3]^ Hypotheses for the mechanism of C5 palsy include spinal cord impairment^[4,5]^ and nerve root involvement,^[6–9]^ but the cause has not yet been determined. Foraminotomy is one of the recommended methods for preventing C5 palsy.^[10–12]^ However, we present a patient who experienced C5 palsy after microendoscopic foraminotomy between the left C5/6 and C6/7 levels, which was not caused by either backward movement of the spinal cord or decompression of the C5 nerve root. This case study was approved by the Ethics committee of Iwai Medical Foundation, and the patient provided informed consent for publication of his case.

## 2 Report of case

### 2.1 History and examination

A 43-year-old man presented with progressive numbness in the left upper limb for 14 months. On neurological examination, deep tendon reflexes and the upper arm motor strength were normal, but we observed paresthesia of the left radial forearm, and the index and middle fingers. Spurling test was positive and the patient had painful numbness extending to the left scapula and the middle finger.

**Figure 1:**
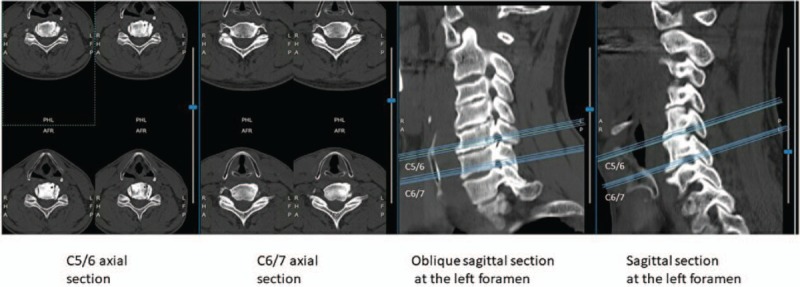
Oblique sagittal computed tomography imaging confirms the presence of left C5/6/7 foraminal stenoses. Axial slices are of the C5/6 and C6/7 levels. Oblique sagittal and sagittal slices are of the left foramen.

**Figure 2:**
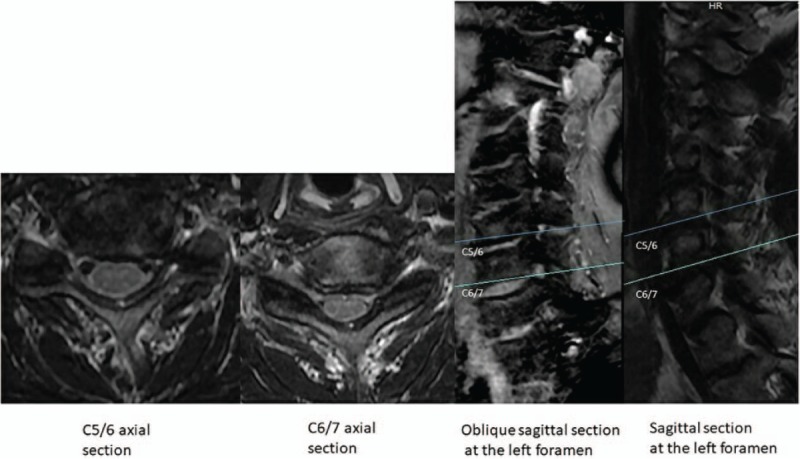
T2-weighted magnetic resonance imaging also demonstrates left C5/6/7 foraminal stenoses. Axial slices are of the C5/6 and C6/7 levels. Oblique sagittal and sagittal slices are of the left foramen.

Computed tomography (CT) imaging (Fig. 1) and magnetic resonance imaging (MRI) (Fig. 2) confirmed the presence of left C5/6/7 foraminal stenoses.

### 2.2 Operation

We performed microendoscopic left foraminal decompressions at C5/6/7 levels to treat the left C6 and C7 radiculopathy. The patient's head was held by a tilted headrest to keep the neck slightly flexed in the prone position. The METRx system (Medtronic Sofamor Danek Inc., Memphis, TN) was used, and an 18-mm incision was made at 1 cm from the left of the tip of the C6 spinous process. The left laminae of C5, C6, and C7 vertebrae were exposed while using a dilator. Keyhole decompression was performed using 3 and 4 mm high-speed diamond drills. The bone, including the medial superior facet, was removed but the flavum was retained.

Intraoperatively, the motor evoked potential (MEP) of the deltoid increased by 20% and that of the brachioradialis decreased by 37%.

### 2.3 Postoperative course

Immediate postoperative motor strength was Medical Research Council (MRC) grade 5/5 for shoulder elevation and elbow flexion on both sides. On the postoperative day 1, we observed weak motor strength of the left deltoid (MRC grade 3/5), left biceps, and left forearm pronator (MRC grade 4/5), while the motor strength of the other muscles was normal. The Spurling test showed improvement and the painful numbness in the fingers disappeared.

**Figure 3:**
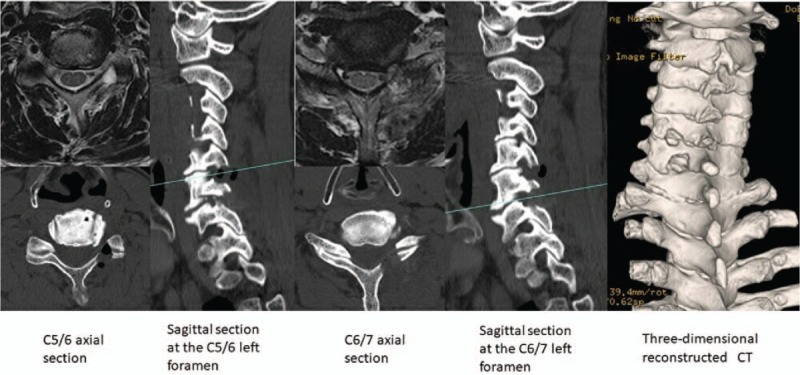
Computed tomography (CT) and magnetic resonance imaging scans (T2 axial slices) after surgery showing sufficient decompression.

CT imaging after surgery showed sufficient bony decompression, and MRI after surgery did not demonstrate insufficient decompression or a high signal change in the spinal cord (Fig. 3). A diagnosis of C5 palsy following C5/6/7 left foraminotomy was made.

After follow-up rehabilitation with muscle strength training and range of motion training, the patient recovered his motor strength completely within 3 months postoperatively.

## 3 Discussion

There are a few reports of C5 palsy occurring after foraminotomy, which did not decompress the spinal cord, cause nerve root impingement, or decompress the C4/5 foramen.^[13]^

Several hypotheses for the cause of C5 palsy have been considered in previous studies.

The first and the commonest hypothesis is the impingement of the tethered C5 nerve root at the inner side of the C4/5 foramina caused by posterior movement of the spinal cord.^[2,4,5]^

The second considered hypothesis is the disruption in blood reperfusion into the spinal cord due to spinal cord decompression^[6,7]^ and the third is drill heat-induced radiculopathy or direct nerve damage caused by the drill.^[8,9]^

In our case, there was no decompression and no posterior movement of the spinal cord causing impingement of the nerve root and disruption in its course. Since we did not decompress the spinal cord, no posterior movement of the spinal cord occurred. Moreover, the yellow ligament or the medial part of the superior articular process where the nerve roots may be impinged by the foramen were removed (Fig. 3).

Similarly, since we did not handle the spinal cord, no disruption in the blood circulation of the spinal cord was identified (Fig. 3).

Video documentation of the surgical procedure was reviewed to rule out direct injury to the nerve root caused by the drill and confirm the absence of decompression; 2 observers confirmed the absence of direct damage to the spinal cord and sufficient decompression. Moreover, the MEP did not drop significantly during the surgery, neither was there a decrease in muscular strength just after surgery.

Conversely, the drill heat could have induced C5 palsy in our case. There have been previous reports of animal studies in which delayed nerve conduction disturbances were caused by heat damage. De Vrind et al^[14]^ reported delayed decrease in motor and sensory function after hyperthermia (30 minutes at 45 °C) of the rat sciatic nerve. Functional damage was apparent 3.5 hours after the heat treatment and complete function loss was achieved 8 hours after the heat treatment. Although it was not a delayed nerve conduction disturbance, Choi et al^[13]^ reported C6 nerve palsy due to thermal injury from drilling for medial facetectomy. Concerning the C5 palsy without C5 nerve root decompression, we hypothesize that the C5 palsy in C5/6/7 foraminotomy could be related to variations in the formation of the brachial plexus^[15,16]^ and can be expected in the post-fixed brachial plexus. The root of the brachial plexus may be formed with small contributions from the anterior rami of C4 or T2. In the prefixed brachial plexus, the origin of the most superior root of the plexus is C4 and that of the most inferior root is C8 or T1. Conversely, in the post-fixed brachial plexus, the origins of the superior and inferior roots are C5/C6 and T2, respectively.

The incidence of prefixed brachial plexus in cadaver studies was 17.5% to 48%,^[15,16]^ whereas the incidence of post-fixed brachial plexus was 2% to 7.5%.^[15]^

The limitation of this case is that since we did not measure the local temperature while using the drill, the cause of C5 palsy in this case is only predictable, not certain. The diagnosis of drill heat-induced C5 palsy is the result of ruling out the generally reported cause of C5 palsy.

The cause of C5 palsy remains unclear amidst much discussion, and this case can provide some advantage in investigating this issue. In this case, the theories of nerve root impingement and disruption of blood circulation as the cause of C5 palsy were not convincing; hence, we considered drill heat-induced C5 palsy. Therefore, prophylactic foraminotomy for cervical posterior surgery should be performed with care, limiting its use in patients who are at risk of developing C5 palsy, because the prophylactic procedure can cause C5 palsy. We must also bear in mind that even without decompression at the C4/5 level, there is a possibility of C5 palsy occurring.

## Author contributions

**Conceptualization:** Masahito Oshina.

**Investigation:** Masahito Oshina, Tomohide Segawa.

**Supervision:** Yasushi Oshima, Sakae Tanaka, Hirohiko Inanami.

**Writing – Original Draft:** Masahito Oshina.

**Writing – Review & Editing:** Masahito Oshina.

Masahito Oshina orcid: 0000-0001-8479-8782.
